# Synergistic Effects of GGBS and Recycled Aggregates on the Tribological, Mechanical, and Fracture Behavior of Polymer Concretes

**DOI:** 10.3390/polym18182183

**Published:** 2026-09-08

**Authors:** Batuhan Aykanat

**Affiliations:** Department of Civil Engineering, Engineering Faculty, Düzce University, 81620 Düzce, Türkiye; batuhanaykanat@duzce.edu.tr

**Keywords:** polymer concrete, recycled concrete aggregate, ground granulated blast-furnace slag, tribological behavior, fracture energy, mechanical properties

## Abstract

While the individual effects of sustainable fillers on cementitious systems are widely known, their combined tribological and fracture behaviors within a polymer concrete matrix remain largely unexplored. Addressing this gap, this study experimentally investigates the physical, mechanical, and tribological characteristics of polyester-based polymer concrete (PC). In addition to reference specimens produced with polyester resin and silica sand, modified mixtures were developed by replacing the silica sand with ground granulated blast-furnace slag (GGBS) and recycled waste concrete aggregate (WC) at various substitution ratios (0%, 5%, 10%, 15%, 20%, and 25%). To evaluate the performance of the developed PCs, parameters including unit weight, water absorption capacity, flexural and compressive strengths, Shore D hardness, surface roughness, acid resistance, Bohme abrasion resistance, and fracture energy were analyzed. Furthermore, temperature variations on the friction surfaces were monitored in real time using a thermal camera during the Bohme abrasion tests. To elucidate the fracture mechanisms, the fractured surfaces were examined via digital microscopy. The quantitative findings indicate that a 25% GGBS replacement optimizes mechanical performance, increasing the compressive and flexural strengths by 21.2% (108.30 MPa) and 30.6% (35.29 MPa), respectively, alongside a 27% improvement in Bohme abrasion resistance. However, this modification significantly increases material brittleness, reducing the fracture energy by 53.3% compared to the reference. Conversely, although incorporating WC offers sustainability advantages, it limits mechanical performance, leading to decreases of up to 9.9% (80.46 MPa) in compressive strength and 15% (22.97 MPa) in flexural strength at a 20% substitution rate. Regarding fracture energy, while the W20 series absorbed more energy than the B25 series, it still remained 47.8% lower than the reference. Additionally, the GGBS-incorporated series demonstrated higher susceptibility to sulfuric acid attack compared to the WC-incorporated series.

## 1. Introduction

Cement, which is primarily used in concrete production, is manufactured using a process that requires a significant amount of energy. In particular, the use of high-carbon-emission fuels such as coal during the clinker production stage makes cement production a significant source of CO_2_ emissions [1,2]. Efforts to reduce CO_2_ emissions by using alternative materials to cement are the subject of ongoing research in the construction industry [3]. As a result, the construction materials sector is continuing to grow, and sustainability requirements for new materials are calling for cheaper and more environmentally friendly products [4]. To meet these needs and solve these problems, the use of polymer concrete has been recommended in recent years. Polymer concrete (PC) is a composite construction material in which polymeric resins are used instead of cement and water [5,6,7].

The industrial implementation of PC traces back to 1958 in the United States, where it was initially utilized for the production of building cladding. In addition to its use in cladding, it has been commonly used as marble for countertops, lavatories, and other sanitary ware, both in the past and today. Its rapid curing, excellent adhesion, light weight, high strength, and durability make polymer concrete an excellent repair material. Thanks to these properties, it is widely used in the maintenance and reinforcement of dams, airports, roads, tunnels, and other structures [8,9].

In PC production, acrylics, epoxy resin, polyurethane resin, unsaturated polyester resin, furan resin, and vinyl ester resin are frequently used as binders [10,11]. The use of industrial waste materials such as fly ash, silica fume, and slag as fillers makes PC an environmentally friendly construction material [12,13]. In addition, PC can sometimes be reinforced with fibers of various types. Chopped glass fiber is among the most commonly used fibers [7,14,15,16].

Studies in the literature have focused on investigating the mechanical and durability properties of PC. Almutairi et al. compared the mechanical behavior of polymer concrete produced using epoxy and polyester resins with that of ordinary concrete. In addition, the study assessed the environmental impacts of PC produced from epoxy and polyester resins. The study results show that PC produced using epoxy and polyester resins is significantly superior to ordinary concrete in terms of flexural strength, compressive strength, and tensile strength. An analysis of the environmental impact assessment results revealed that polymer concrete made with polyester resin produces lower emissions of NO_2_ and SO_2_ in particular, and is more consistent with environmentally friendly production criteria [17]. In a study where sawdust and chopped PET bottle waste were used as aggregate substitutes in varying proportions, the effect of using waste as an aggregate substitute on density, workability, and compressive strength was investigated. The results showed that using waste as an aggregate substitute reduced the density of polymer concrete in all mixtures [18]. In another study on waste materials, the effect of using argillaceous powder, calcareous powder, marble powder, and fly ash as waste materials on the behavior of polymer concrete was investigated. According to the results of the study, the addition of calcareous powder and fly ash improved the mechanical properties, while the addition of argillaceous powder material caused a decrease in mechanical properties compared to the reference sample [19]. A comprehensive review of research on fracture mechanics in polymer concrete has been presented. The study discusses factors that influence the fracture parameters and fracture patterns of polymer concrete, including resins, aggregate, and fillers, specimen sizes and shapes, crack depth, and crack angles [20]. In a study carried out by Hashemi et al., the fracture mechanics and flexural strengths of polymer concrete produced using unsaturated polyester resin were determined after the specimens were aged for 1, 3, 6, and 12 months in sulfuric acid and alkaline environments. The results indicated that the polymer concrete degraded in both environments, and that this degradation significantly affected its fracture mechanics and flexural strength behavior [21]. A general review of the literature reveals that the properties of resins, aggregates, and fillers, as well as the potential use of sustainable materials, are topics of interest. Although research on polymer concrete is ongoing, the use of blast furnace slag—an industrial by-product—and ground waste concrete aggregate remains very limited. For this reason, in order to make a significant contribution to the existing literature and to evaluate waste materials, ground granulated blast-furnace slag (GGBS) and recycled waste concrete aggregate (WC) was utilized. In this way, by utilizing GGBS, an industrial waste material, the potential contributions it can make to the mechanical, physical, and durability properties of polymer concrete will be examined in detail. In addition, an alternative method for reusing concrete obtained from construction debris by grinding it using a simple process will be investigated. In this way, by offering an alternative perspective on the reuse of waste concrete, a contribution will be made to the construction industry’s environmental sustainability goals.

The significance and novelty of the current study lie in comprehensively investigating the combined effects of substituting silica sand with different proportions of GGBS and recycled concrete aggregates (WC) in a matrix structure where the polyester resin content is kept constant. Within the scope of the study, tests for unit weight, water absorption, acid resistance, flexural strength, compressive strength, abrasion resistance, and fracture energy were conducted. Although the existing literature has focused on the mechanical performance of polymer concrete (PC), the relationship between the tribological behavior and thermal characterization of these composite materials with waste substitutes has not yet been fully elucidated. In this context, the study presents an innovative methodology that includes the monitoring of instantaneous temperature changes on the friction surface during the Bohme abrasion test using a thermal camera and a comparative analysis of the abrasion amounts. Additionally, fracture energy data determined through three-point bending tests on notched beams will provide critical insights into the material’s ductility and crack propagation resistance, thereby contributing to the literature. In short, this study presents a novel engineering approach to the design of polymer concrete by combining physical, mechanical, and durability parameters using innovative methods.

## 2. Materials

### Materials and Mixture Details

In this study, a casting-type polyester resin obtained from Eskim Kimya (Eskişehir, Türkiye) was used as a binder in the production of polymer concrete; cobalt octoate was used as an accelerator and methyl ethyl ketone peroxide (MEK-P) as an initiator to control the curing reaction. Some properties of polyester resin are given in Table 1.

Silica sand (SS) with an AFS grain-size distribution of 30–35 was used as aggregate in the production of polymer concrete.

**Figure 1:**
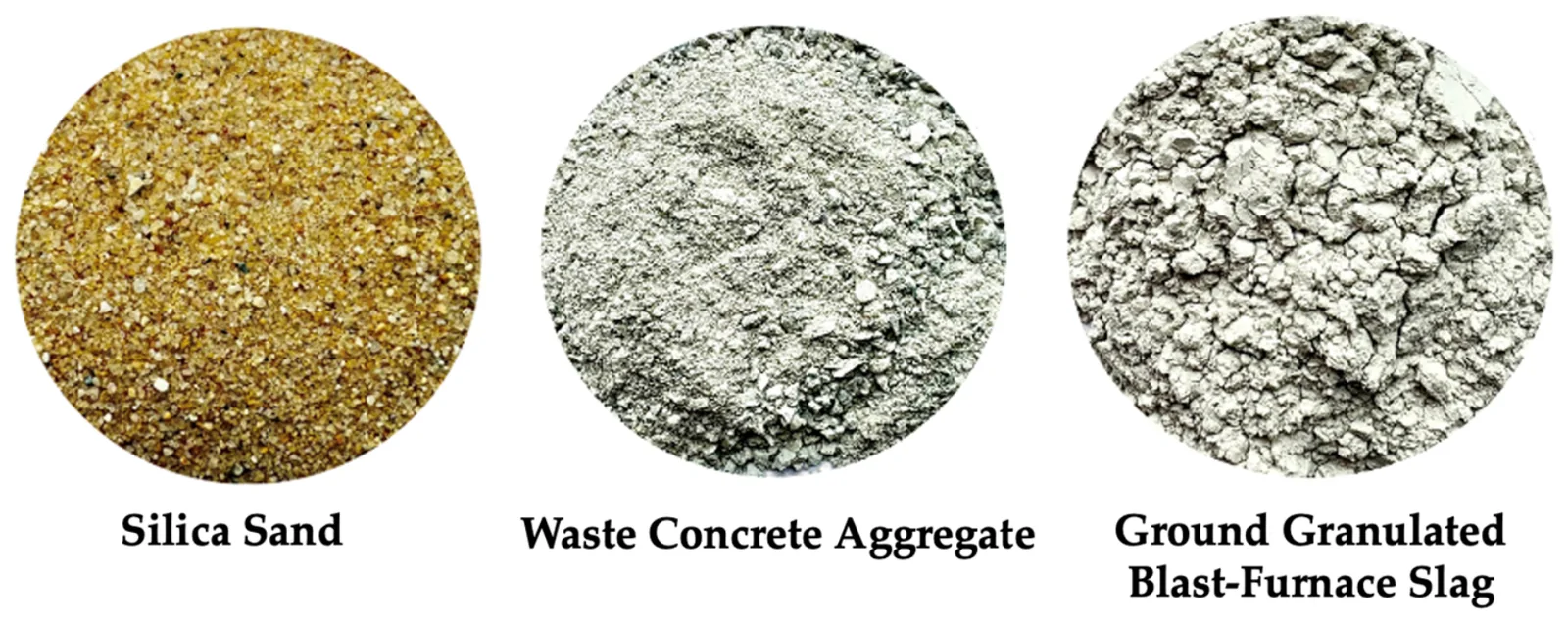
Silica sand, WC and GGBS.

In the second stage of the study, silica sand was substituted on a volumetric basis at varying replacement levels (0% (REF), 5%, 10%, 15%, 20%, and 25%) with ground granulated blast-furnace slag (GGBS) and recycled waste concrete aggregate (WC). GGBS, an industrial waste product used as a substitute for silica sand, was obtained from OYAK Cement Facilities (Bolu, Türkiye). GGBS was used to fill the voids between the coarse grains of silica sand to obtain a denser matrix structure. As a mechanical pretreatment, the WC used in the study was obtained by crushing waste concrete using a jaw crusher in the laboratory and sieving it through a 2 mm mesh. No additional chemical or thermal treatments were applied prior to its incorporation into the polymer concrete. Waste concrete (WC) exhibits a more porous and irregular structure compared to the relatively dense and uniform structure of silica sand. For this reason, different replacement ratios of GGBS and WC were used to determine the feasibility of incorporating them into polymer concrete. Images of silica sand, GGBS, and WC are given in Figure 1.

## 3. Methods

**Figure 2:**
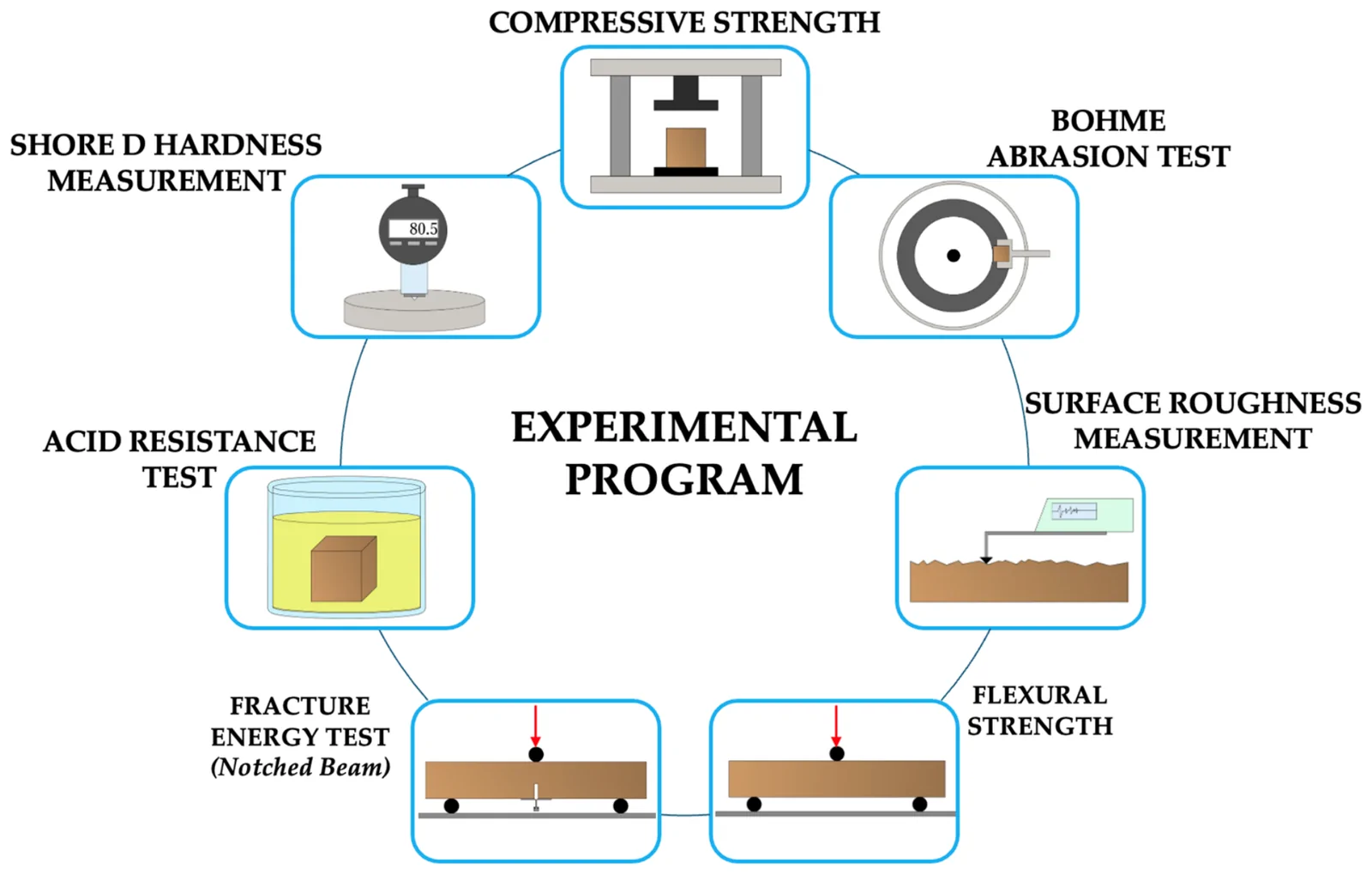
Schematic representation of the experimental program.

The experimental setup used in the study is shown schematically in Figure 2.

### 3.1. Preparation of Mixtures

**Figure 3:**
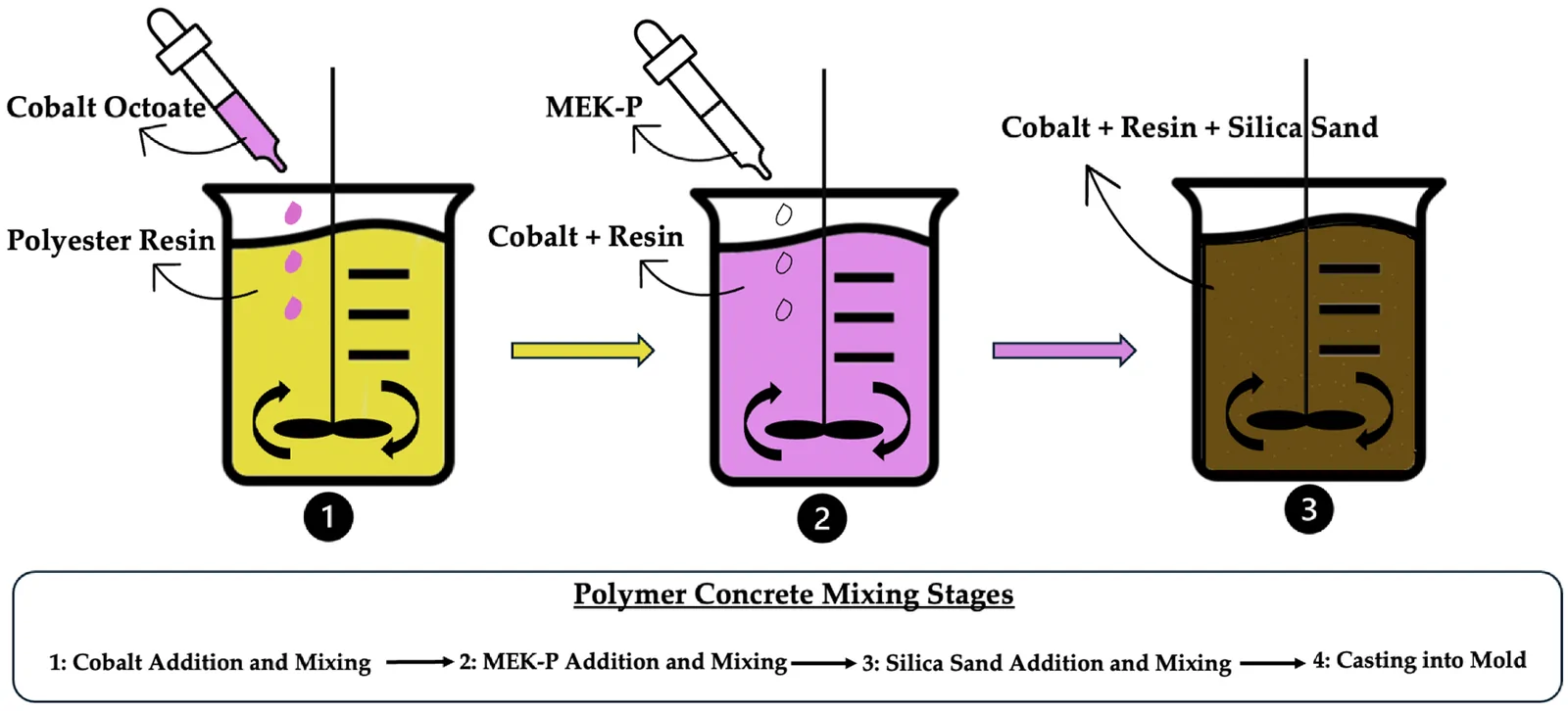
Mixing procedure.

In the production of polymer concrete, the polyester resin content, constituting the binder phase, was maintained at 20% by weight, whereas the accelerator (cobalt octoate) and initiator (MEK-P) contents were adjusted to 0.3% and 1% by weight of the polyester resin, respectively. During the preparation of the mixtures, cobalt octoate was first added to the polyester resin and mixed thoroughly to achieve a homogeneous composition. Subsequently, methyl ethyl ketone peroxide (MEK-P) was introduced, and mixing continued to initiate the reaction. The aggregate phase (SS, SS + WC, SS + GGBS) was then added, and mixing was maintained until a uniform mixture was obtained. The resulting mixture was placed into molds using a tabletop vibrator and demolded after 24 h. The specimens were then cured in an oven at a constant temperature of 65 °C for 6 h to ensure complete polymerization. The mixing stages are shown in Figure 3.

### 3.2. Experimental Study

In order to determine the fundamental physical and mechanical properties of the produced polymer concretes, unit weight, water absorption, acid resistance, Shore D hardness measurements, surface roughness, flexural strength, and compressive strength tests were conducted. Samples that show optimal results across all series will be selected, and Bohme abrasion tests and fracture energy tests will be conducted on these samples. The specimen dimensions for the tests are shown in Table 2.

### 3.3. Flexural Strength and Compressive Strength

**Figure 4:**
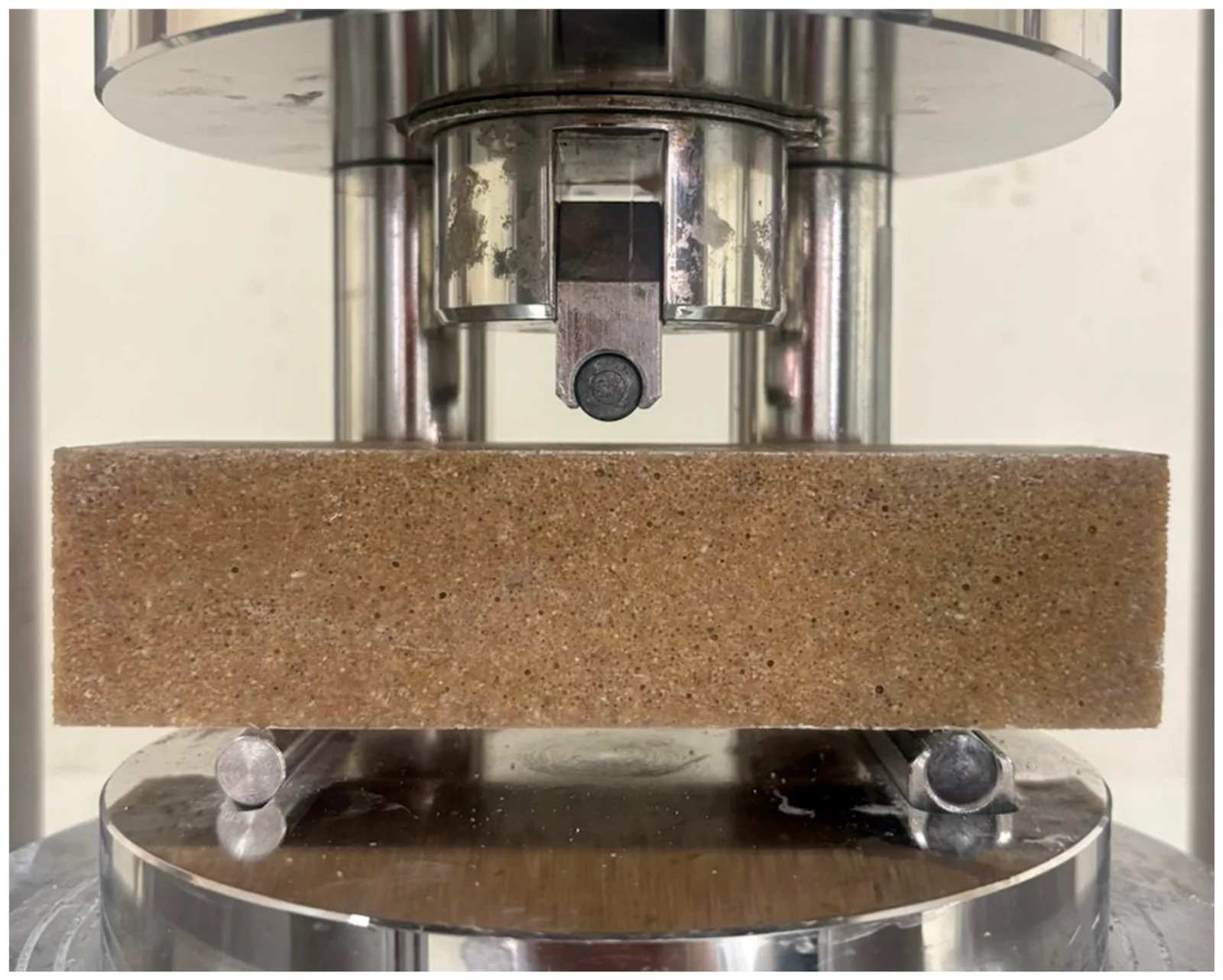
Three-point bending test.

Compressive strength and flexural strength tests were performed on the polymer concrete specimens in accordance with the TS EN 196-1 standard [22]. The three-point bending test setup is shown in Figure 4.

### 3.4. Shore D Hardness Test

In polymer concrete, Shore D hardness testing serves to evaluate surface hardness and stiffness characteristics, while also providing an indirect indicator of the degree of polymerization. Accordingly, Shore D hardness measurements were conducted on the specimens. Shore D hardness was measured using a digital hardness tester (Loyka, Istanbul, Türkiye) capable of measuring between 0 and 100. The highest value recorded from measurements taken from five different regions of the sample was recorded as the hardness value.

### 3.5. Acid Resistance Test

Furthermore, to simulate aggressive industrial environments where polymer concrete may be exposed to acidic media (e.g., sewage systems, chemical plants, and industrial wastewater structures), the acid resistance of the specimens was evaluated by immersing them in a 10% sulfuric acid solution for 28 days. This test was conducted to assess the durability and degradation behavior of the material under severe chemical attack conditions. Following the exposure, mass loss, Shore D hardness variations, and compressive strength values were measured to assess the degradation behavior and durability of the material.

### 3.6. Surface Roughness Measurements

Surface roughness measurements of polymer concretes were carried out to determine the surface quality, homogeneity, and microstructural irregularities that may occur depending on the production process. Surface roughness measurements were carried out using a portable contact profilometer (Mahr, Göttingen, Germany), compliant with the DIN EN ISO 21920 standard [23], with profile filtering performed according to ISO 16610 [24]. The device operates based on a stylus-type measurement principle, in which a diamond-tipped probe traverses the surface and records micro-scale profile variations, enabling the determination of standard roughness parameters such as Ra, Rz, and Rt. Ra refers to the overall arithmetic mean of all surface irregularities, Rz refers to the average difference between the five highest peaks and the five deepest valleys, and Rt refers to the maximum total distance between the highest peak and the deepest valley across the entire measurement area.

### 3.7. Bohme Abrasion Test

Bohme abrasion tests were carried out on the specimens that exhibited superior mechanical performance. The Bohme abrasion test was performed in accordance with the EN 14157 standard [25]. The amount of abrasion was determined by converting the measured mass loss to volume loss using the sample density. In addition to determining the amount of wear, the thermal variations occurring at the friction surface were monitored using a thermal camera, and the recorded temperature distributions were evaluated in relation to the wear behavior.

### 3.8. Fracture Energy

**Figure 5:**
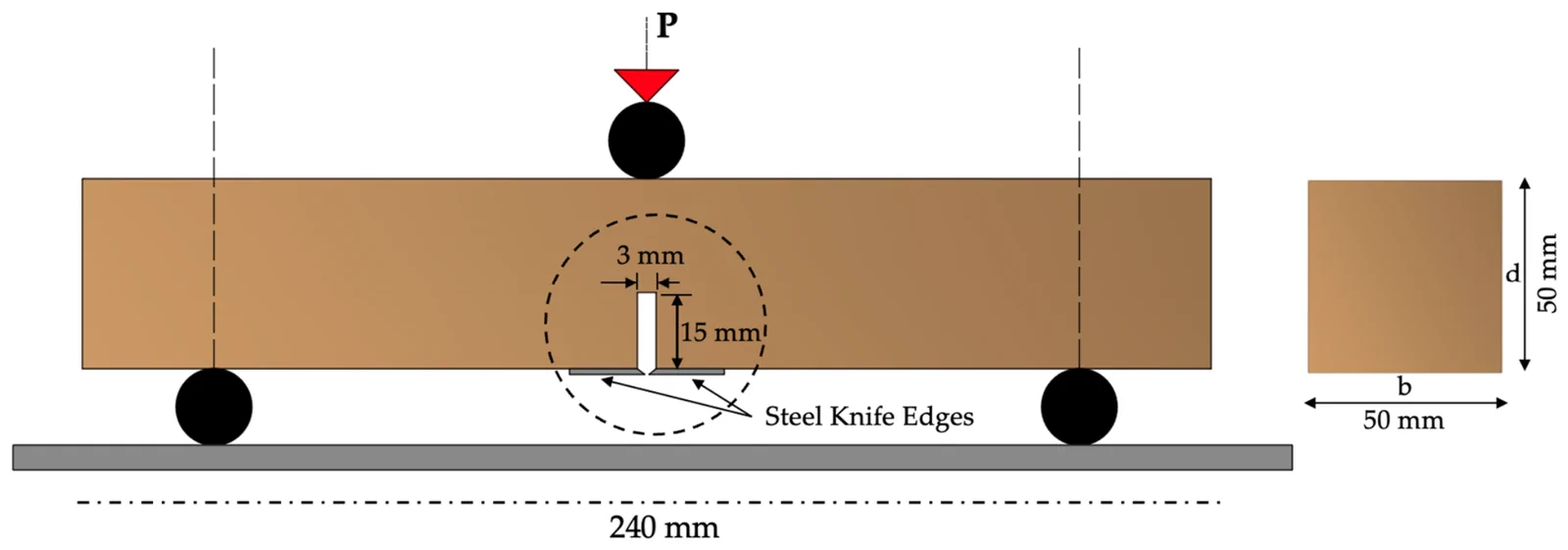
Notched beam.

**Figure 6:**
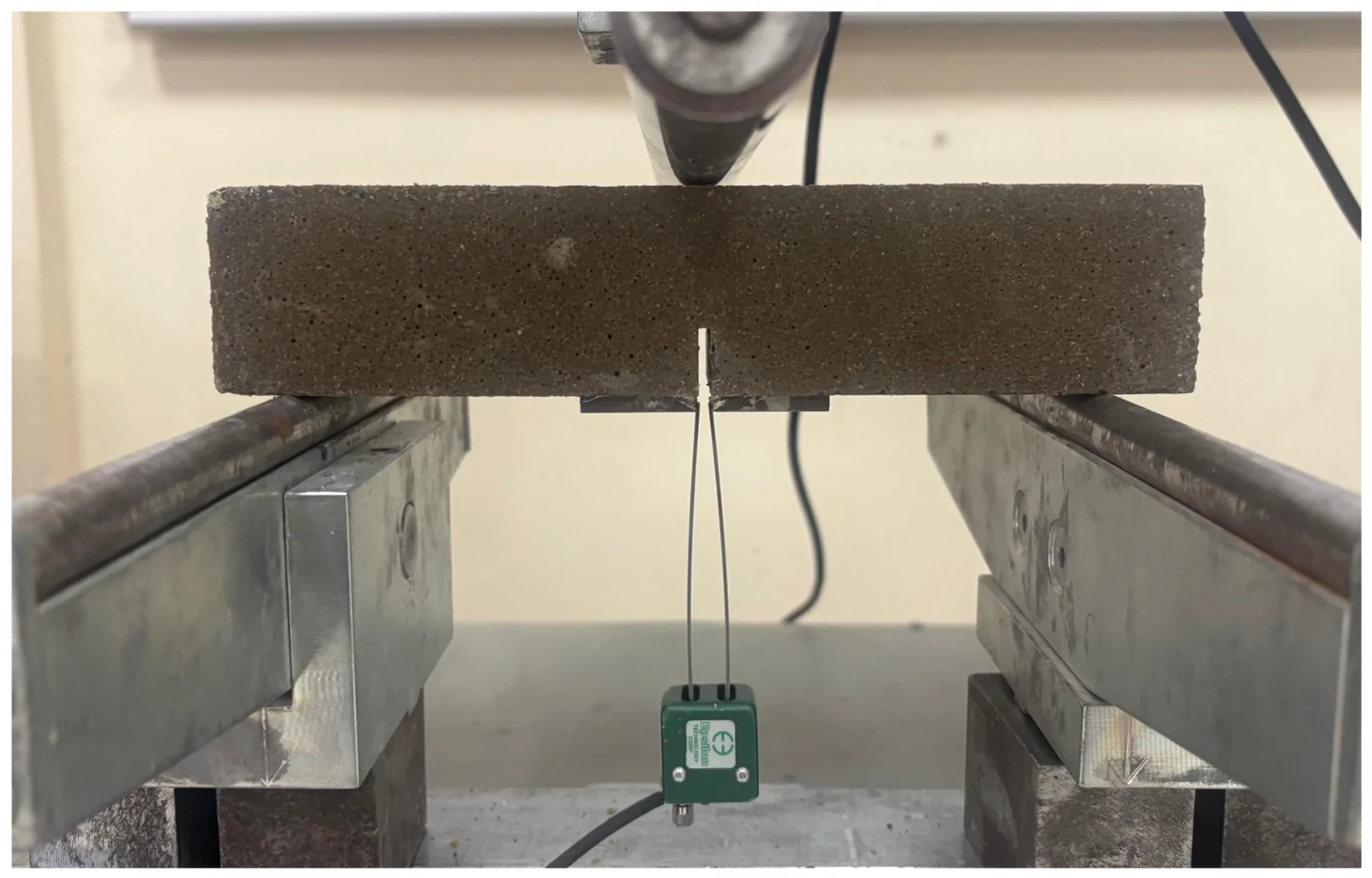
Test setup.

From the mixtures that stood out in terms of mechanical performance, notched beam specimens with dimensions of 50 × 50 × 240 mm were prepared (Figure 5 and Figure 6). The notch length was chosen as 15 mm. A three-point bending test was performed on these notched beams to determine the fracture energy using a deformation-controlled electromechanical testing machine (Besmak, Ankara, Türkiye). The loading rate of the test was determined as 0.009 mm/min, and the end of the test was determined as a 95% reduction in peak load. CMOD (clip-on gauge) was measured using a measuring instrument placed in the notch opening. The fracture energy was calculated using Equation (1), as suggested by RILEM [26]:

(1) $$G_{f}=\frac{\left(W_{0}+mg\delta \right)}{A},$$

where W_0_ denotes the area under the load–CMOD curve (N·mm); m represents the self-weight of the specimen between the supports (kg); g is the gravitational acceleration (m/s^2^); δ corresponds to the maximum displacement (m); and A defines the effective fracture area (m^2^), calculated as b(d-a_0_), with b and d representing the width and height of the beam, respectively. In addition, images were taken of the surfaces of the samples that fractured as a result of the fracture energy test using a digital microscope, and examined in detail.

## 4. Results and Discussion

**Figure 7:**
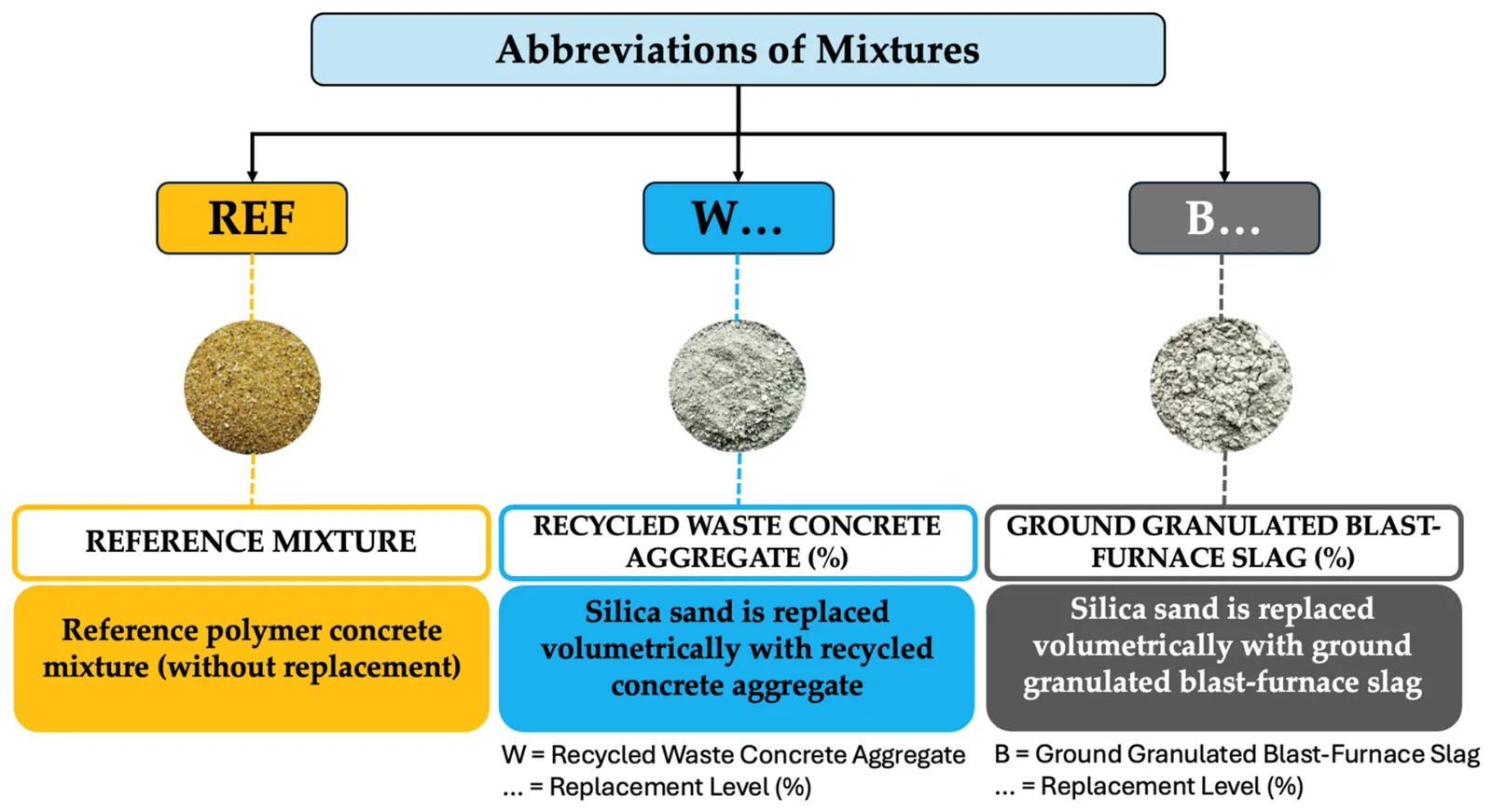
Abbreviations of mixtures.

The abbreviations used to describe polymer concrete in this study are given in Figure 7.

### 4.1. Unit Weight, Compressive Strength, and Flexural Strength

**Figure 8:**
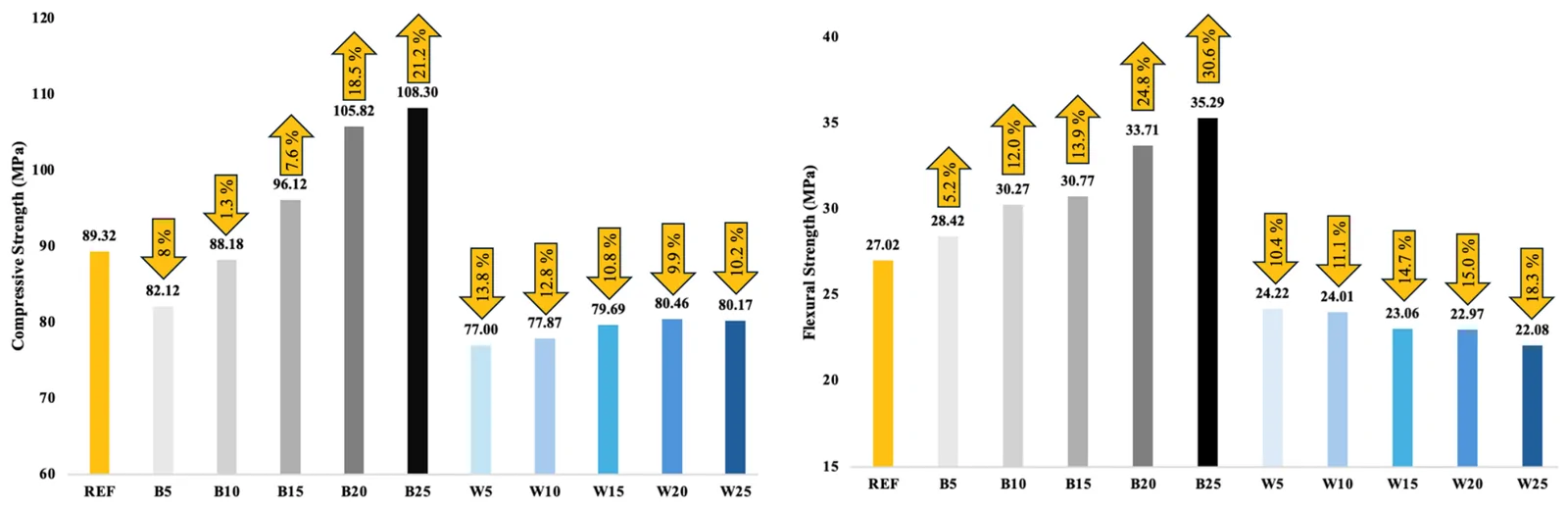
Graphical representation of the compressive strength and flexural strength test results.

The results of unit weight, compressive strength, and flexural strength tests performed on the polymer concrete are given in Table 3. A graphical representation of the compressive strength and flexural strength test results is given in Figure 8.

The experimental results obtained (Table 3 and Figure 8) clearly demonstrate that the type of filler used in polymer concrete mixtures is a determining factor for both physical and mechanical performance. In the B series specimens containing GGBS, it was observed that with increasing filler content, the unit weight increased from 1.84 g/cm^3^ to 2.06 g/cm^3^, and correspondingly, the compressive strength increased from 82 MPa to 108 MPa, while the flexural strength increased from 28 MPa to 35 MPa. This indicates that GGBS reduces the void ratio through its fine filler effect, leading to a denser internal structure and improving the adhesion between the polymer matrix and the aggregate. In contrast, although a limited increase in unit weight was observed in the W series specimens containing WC, the compressive strengths remained within the range of 77–80 MPa, and the flexural strength decreased with increasing additive content, reaching 22 MPa. This suggests that the old mortar phase and microcracks present on the surface of recycled aggregates hinder the formation of an effective interfacial bond with the polymer matrix and lead to early crack initiation, particularly under tensile/flexural behavior. As a result, it is concluded that GGBS provides significant improvements in both the strength and structural integrity of polymer concretes, whereas WC, although advantageous in terms of sustainability, has a limiting effect on mechanical performance. The findings are consistent with studies in the literature reporting that the mechanical performance of polymer concretes is significantly controlled by the particle size of the filler, its void-filling capacity, and the quality of adhesion at the polymer matrix–aggregate interface. In essence, various studies have demonstrated that resin and filler materials reduce voids in polymer concrete, resulting in a more compact structure and enhancing mechanical properties [6,17,27].

### 4.2. Water Absorption Test

The results of the water absorption tests conducted on the PC samples on days 1, 7, 14, and 28 demonstrated that all series exhibited completely impermeable behavior and did not absorb water. These results confirm that the polymer matrix completely covers the aggregates and forms an impermeable structure. The zero-absorption rate observed from day 1 to day 28 confirms that the bond structure of the polymer matrix increases during the curing phase, reaching a significant density and thereby preventing capillary water ingress. Similar water absorption tests conducted on polymer concrete have also concluded that PC does not absorb water or that its water absorption value is negligible (<1%) [3,28]. These findings corroborate the results obtained in this study. It is supposed that this impermeability of PC will make it more resistant to cyclic effects, such as freeze–thaw.

### 4.3. Surface Roughness

**Figure 9:**
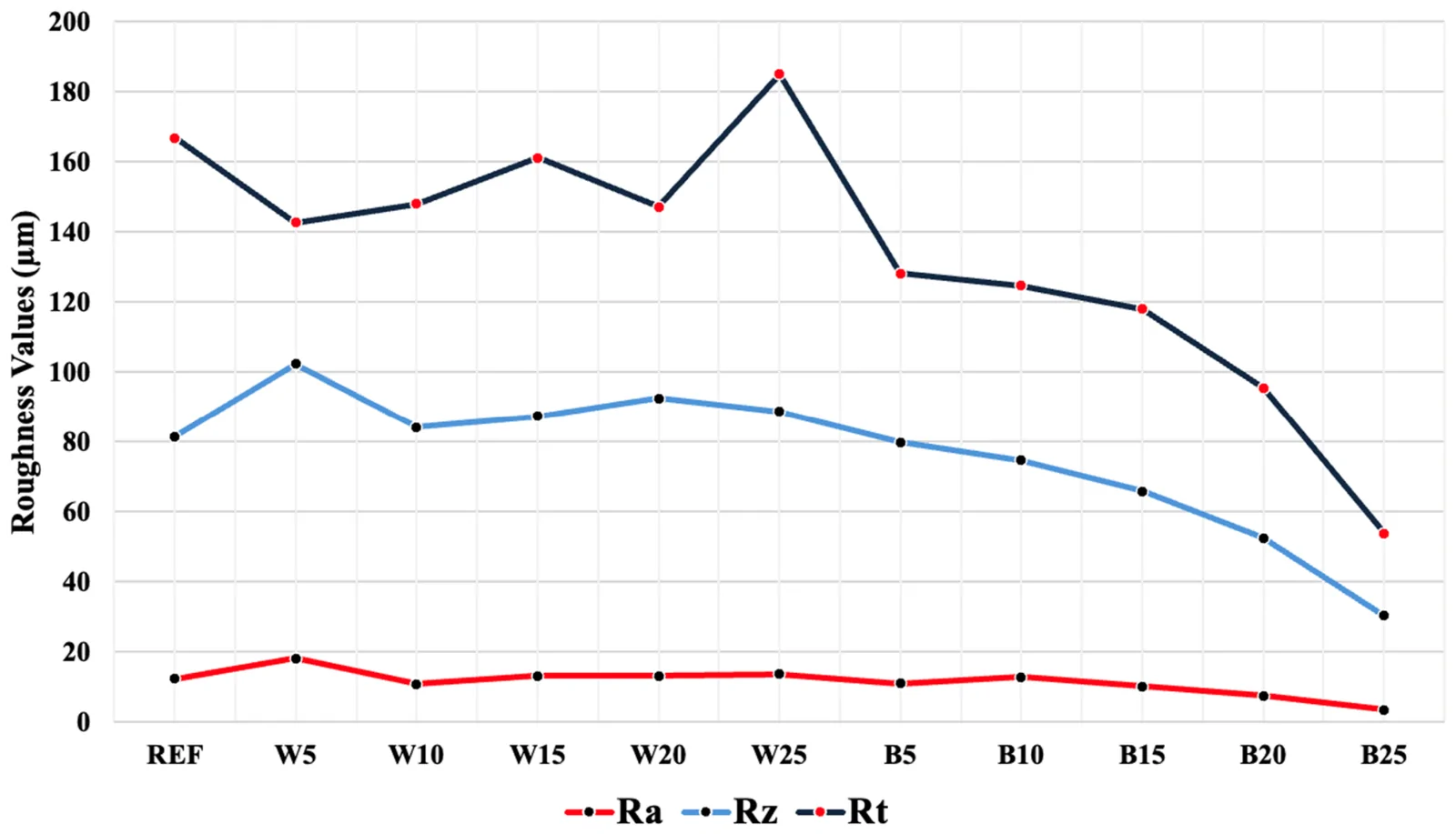
Surface roughness values. Ra: The overall arithmetic mean of all surface irregularities. Rz: The mean difference between the five highest peaks and the five deep valleys. Rt: The maximum total distance between the highest peak and the deepest valley.

Surface roughness test results are given in Figure 9.

The obtained surface roughness results (Figure 9) clearly demonstrate that the surface topography of polymer concrete is directly governed by the aggregate particle-size distribution and filler content. Compared to the reference sample, the increase in Ra, Rz, and particularly Rt parameters observed in the W series specimens, in which the WC content was gradually increased, indicates the development of discontinuities such as pronounced aggregate protrusions, insufficient matrix coverage, and localized surface voids. In contrast, the systematic decrease observed in all roughness parameters in the B series, where the fine filler content was increased, can be attributed to the effective filling of voids, enhanced continuity of the polymer matrix, and the formation of a denser surface structure. In particular, it was found that a 25% GGBS substitution (B25) maximizes surface quality by reducing the Ra value by approximately 72% compared to the reference. This demonstrates that voids are effectively filled due to GGBS’s more compact grain structure and its high interfacial compatibility with polyester resin. On the other hand, waste concrete (W series) shows an increasing trend regarding surface roughness values compared to the reference sample, due to the material’s heterogeneous grain structure. In particular, the changes in Rz and Rt values indicate that the waste concrete forms a more irregular profile geometry within the polymer matrix. From an engineering perspective, the W series specimens provide an advantage in terms of mechanical adhesion due to increased surface roughness; however, they are considered to have the potential to create disadvantages in terms of local stress concentrations and wear initiation due to their high Rt values. In contrast, the B series specimens, with lower roughness and higher surface integrity, exhibit characteristics that may provide superior performance in terms of wear resistance and impermeability; however, this may necessitate additional surface treatment to enhance adhesion in certain applications. Consequently, optimizing surface roughness in polymer concretes requires careful design of the coarse aggregate–fine filler balance depending on the targeted performance criteria; it is understood that Ra alone is not a sufficient parameter, and that a comprehensive evaluation together with Rz and particularly Rt is essential.

### 4.4. Shore D Hardness and Acid Resistance Tests

**Figure 10:**
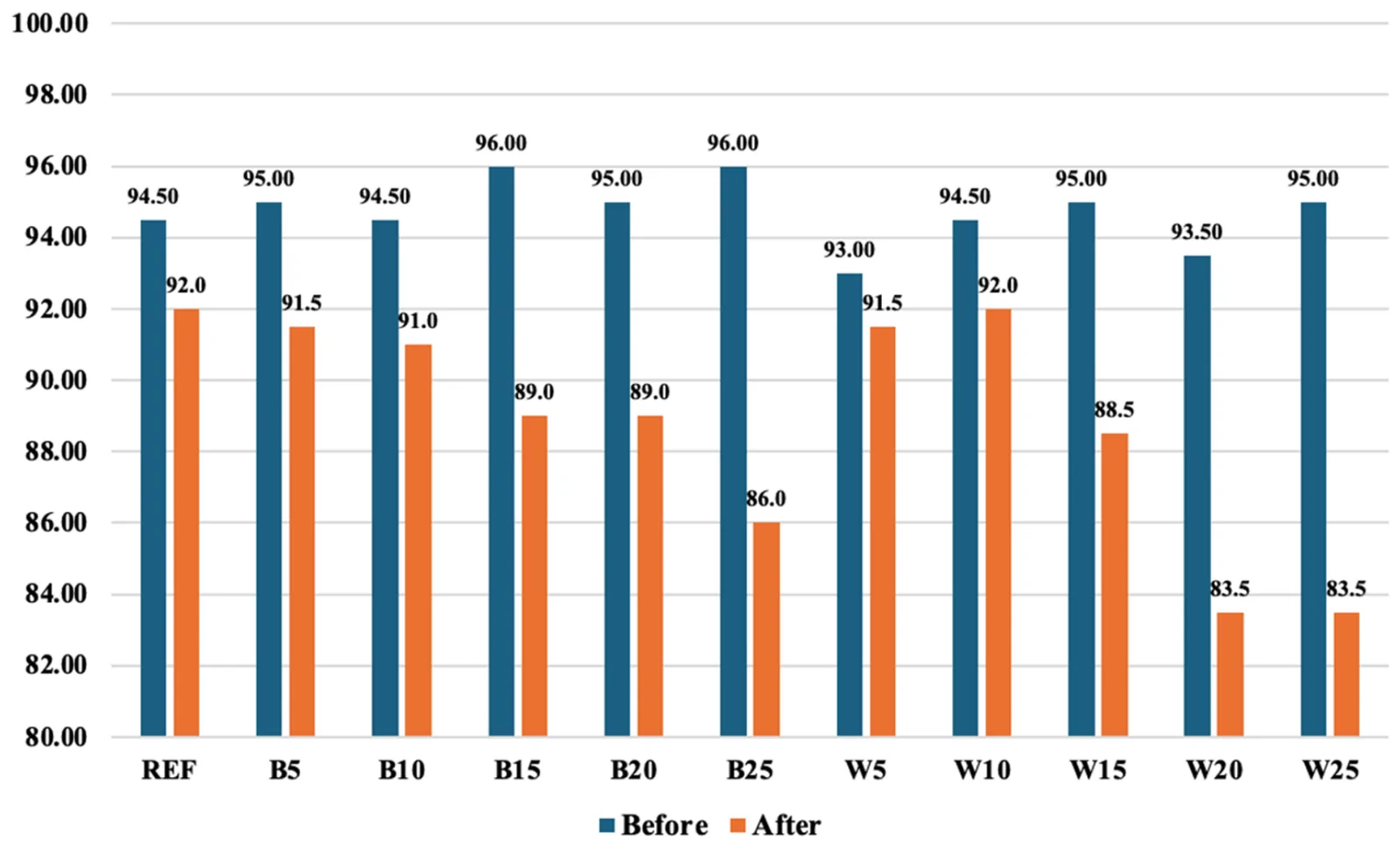
Shore D hardness measurements.

**Figure 11:**
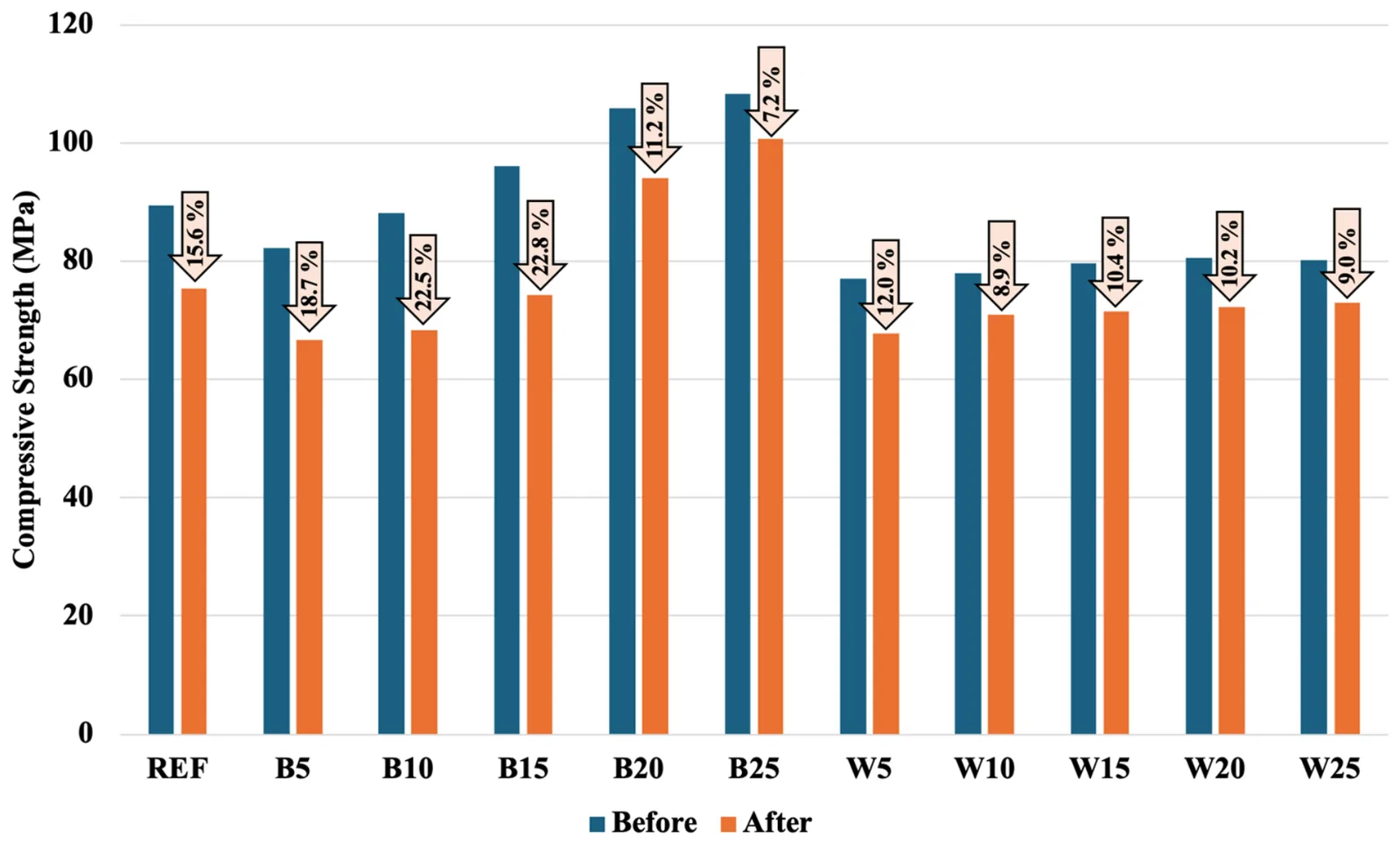
Changes in compressive strength.

The Shore D hardness measurements and compressive strength values obtained at the end of the 28-day acid resistance test (before/after) are shown in Figure 10 and Figure 11, respectively. In the mass loss measurements conducted at the end of the acid resistance test, no mass loss was detected in the PC samples.

The Shore D hardness directly measures the penetration resistance of the polymer concrete surface. As shown in Figure 10, the fact that the initial values are 90+ in all series indicates that the curing process of the polyester resin used has largely been completed. An analysis of the results of the Shore D hardness test conducted on PC samples following the acid resistance test revealed that losses in surface hardness became more pronounced as the replacement ratios of GGBS and WC in the mixtures increased. The greatest decreases in Shore D measurements occurred in the B25 (approx. 10%) and W25 (approx. 12%) series, respectively. This situation can be explained by the chemical interaction between the reactive calcium components (CaO) in the GGBS and WC, which are used as substitutes for inert silica sand, and the sulfuric acid solution. It is considered that during Shore D testing, the acid attack dissolves these calcium-based mineral fillers encapsulated by the polymer matrix, creating micro-scale voids and localized pitting in the surface layer; when the testing probe encounters these areas, lower results are obtained.

When the compressive strength values were examined following the acid resistance test, a decrease ranging from approximately 7.2% to 23% was observed in all samples. The greatest decrease was observed in the B15 series (approx. 23%). In the literature on polymer concrete durability, chemical degradation is typically associated with matrix dissolution and a subsequent increase in interconnected porosity [28]. However, the polyester matrix in this study remains chemically inert against acidic attacks, successfully preventing macroscopic mass loss and capillary water ingress. The significant loss in compressive strength is therefore attributed to microscopic structural degradation rather than physical erosion. The acid reacted with the reactive additives (GGBS, WC) in the surface layer, creating localized internal expansion stresses. It is believed that microcracks formed under these internal stresses and that the specimens sustained damage as a result. It is also considered that water absorption did not occur because the resulting damage was too small to allow capillary water absorption. WC consists of a matrix that has already undergone hydration. Consequently, even when ground, the proportion of CaO and reactive calcium components in its composition is much lower than that of GGBS, an industrial waste. When the decrease in compressive strength of the series replaced by WC and those replaced by GGBS is evaluated together, a maximum reduction of approximately 12% is observed in the W series, while a maximum reduction of approximately 23% is observed in the B series. For this reason, it is considered that the W series is less affected by sulfuric acid attacks.

Based on the mechanical and physical test results, the B25 and W20 series, which yielded optimum results, were selected and subjected to Bohme abrasion and fracture energy tests along with the reference sample.

### 4.5. Bohme Abrasion Tests

**Figure 12:**
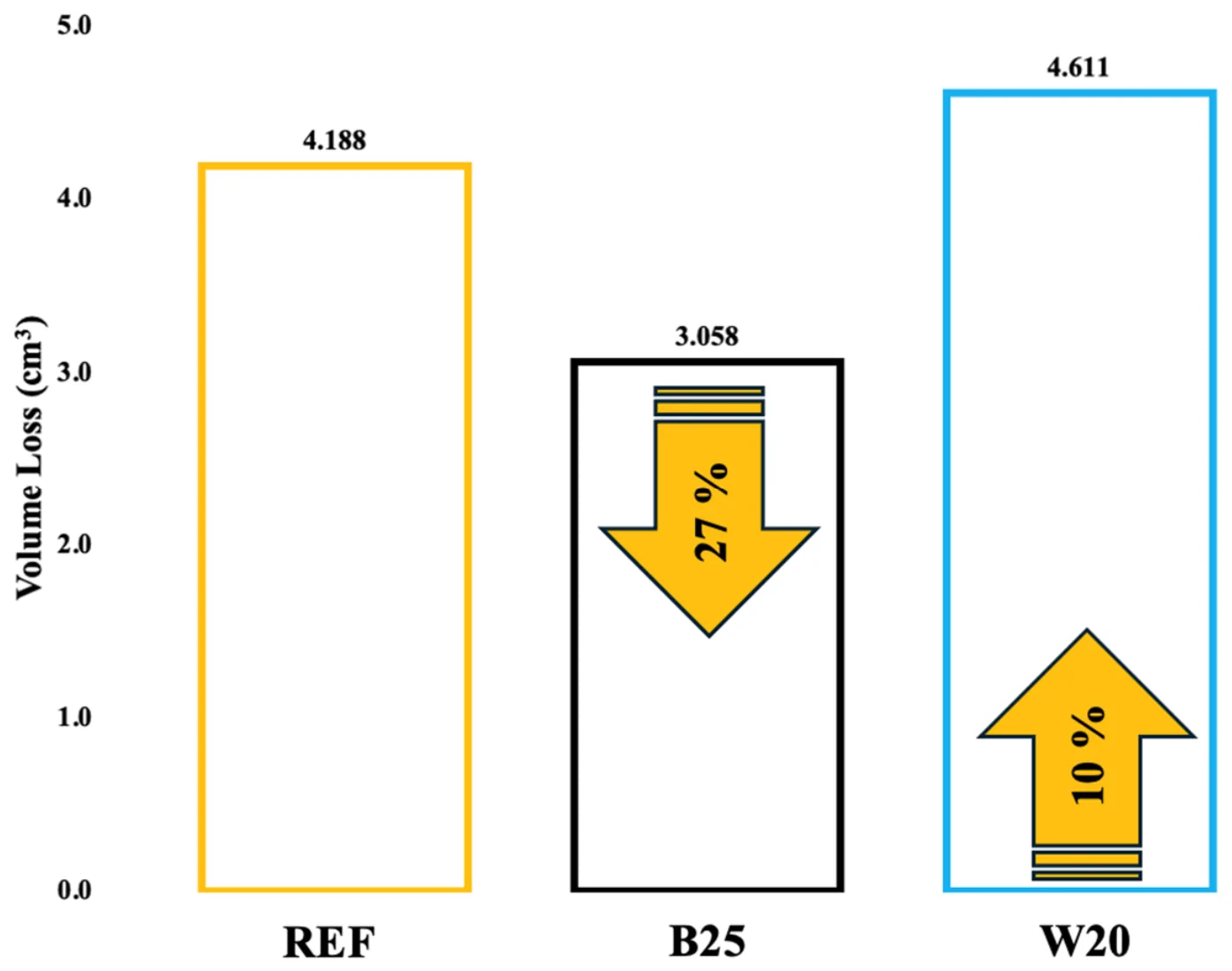
Bohme abrasion test result.

**Figure 13:**
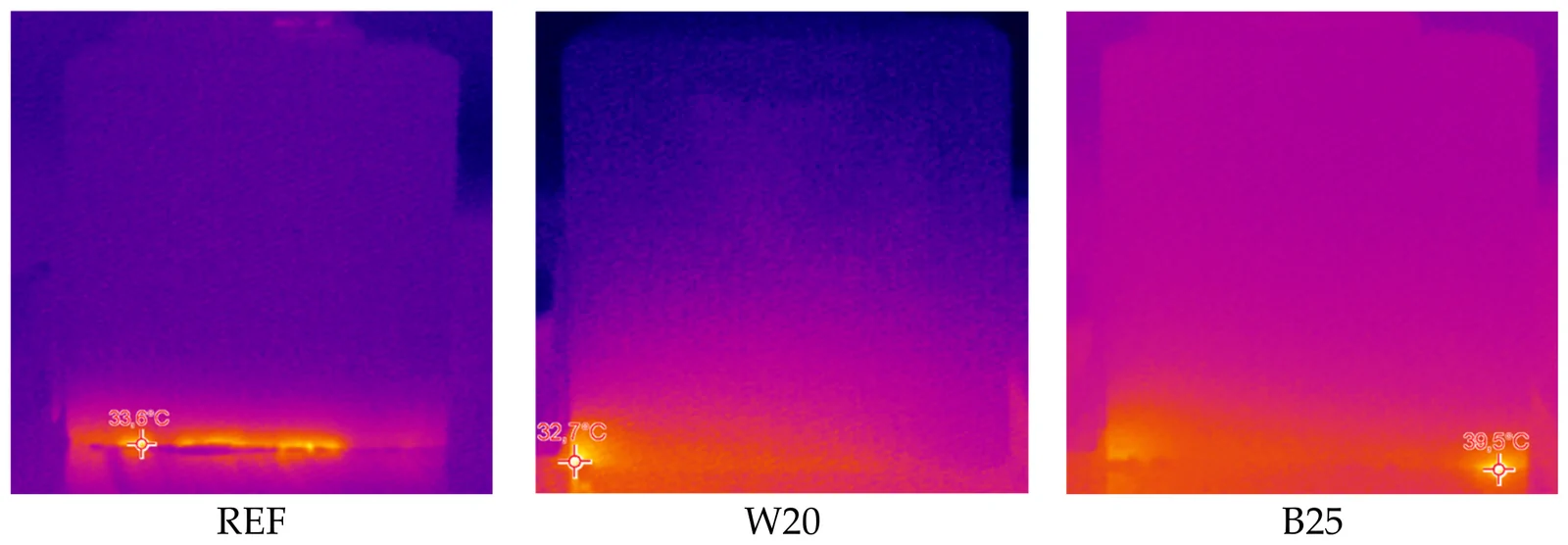

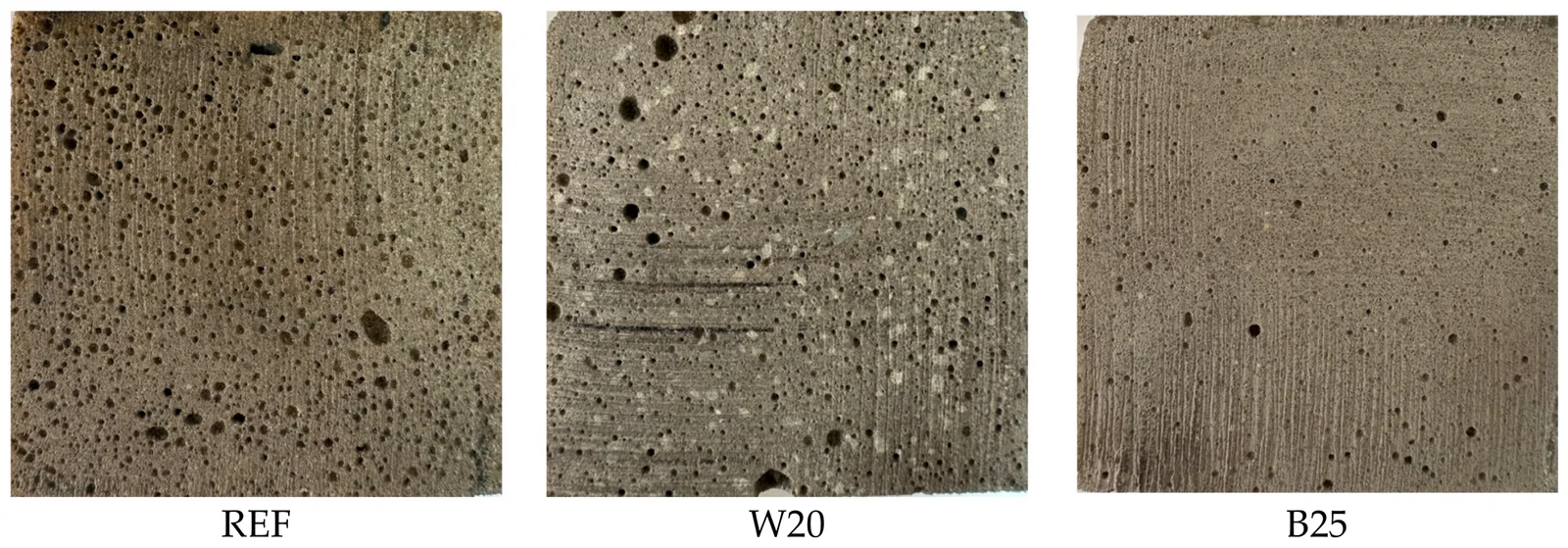
Thermal camera images and wearing surface.

**Figure 14:**
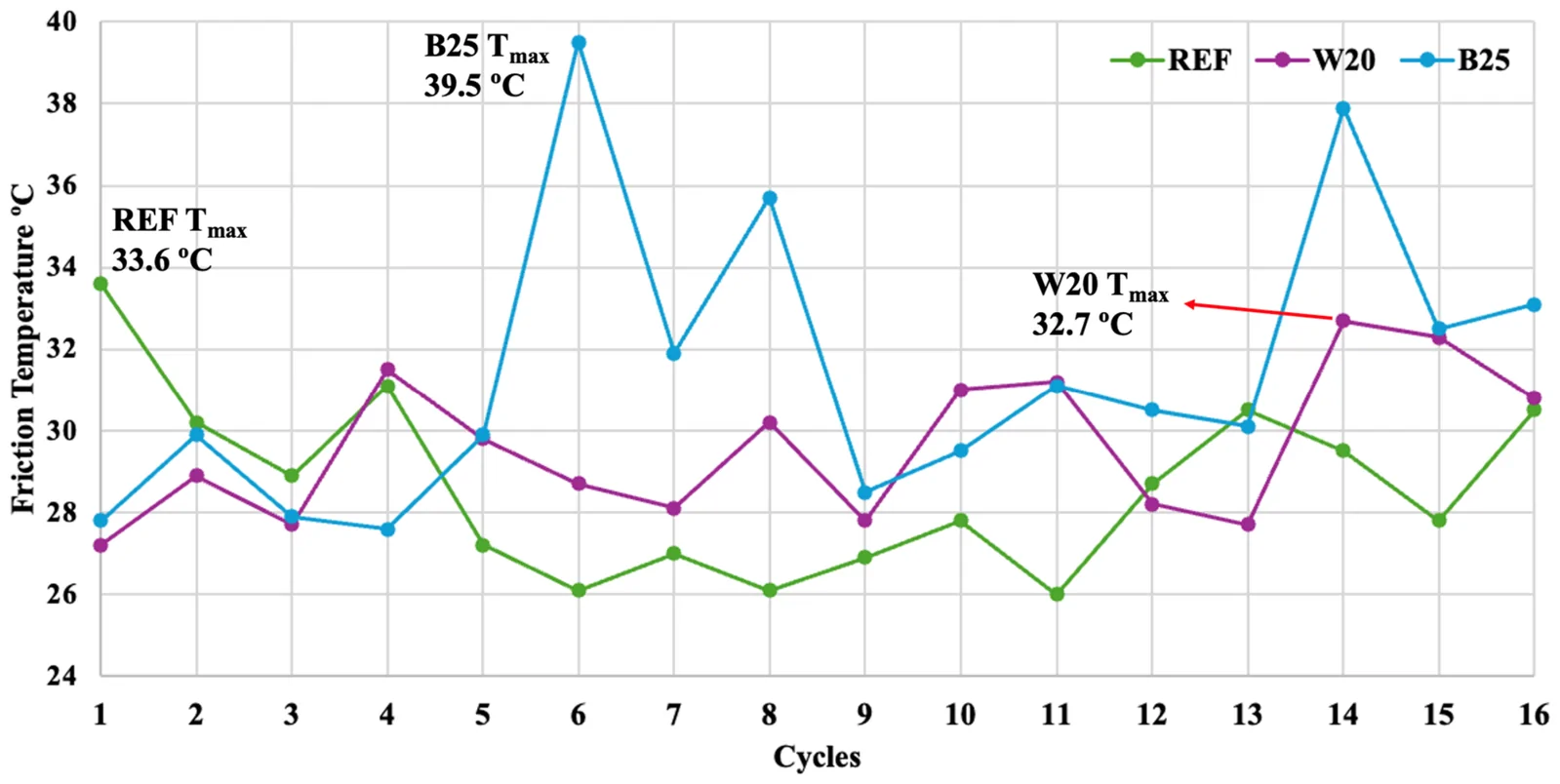
Friction temperatures.

The Bohme abrasion test results are given in Table 4 and Figure 12, the thermal camera images taken during the test are shown in Figure 13, and the graph showing the maximum temperatures measured during the test is also presented in Figure 14.

When the volumetric loss results are examined, it is seen that the B25 series lost 27% less volume compared to the reference. This confirms that GGBS creates an abrasion-resistant surface texture with a high void-filling potential in polymer concrete. These results show that GGBS not only smooths the surface (previous surface roughness test results) but also significantly increases the structural abrasion resistance of the material. The W20 series had the highest volumetric loss at 4.661 cm^3^. In the W series, it is thought that the interfacial bond between WC and the polymer matrix is weak; therefore, the aggregates detach more easily from the surface during abrasion cycles.

When Figure 12 and Figure 14 are examined, it can be seen that the B25 series, despite having the lowest volume loss, reached the highest average temperature (31.46 °C) and the highest peak temperature (39.50 °C). This is due to the B25′s much more compact structure and higher density (2.06 g/cm^3^) compared to the reference material. Because the surface is smooth, the actual contact area between the abrasive disk and the polymer concrete surface has increased. The increased contact area has led to the conversion of friction energy into heat over a larger surface area and, due to the material’s high density, to greater heat retention within the material. The W20 series, which has the lowest standard deviation (1.80), exhibited thermally stable (lowest fluctuation) behavior. The fact that the W20 has the highest volumetric loss (4.661 cm^3^) indicates that particles are continuously breaking away from the surface during friction. These detached particles may have prevented the temperature from rising excessively by carrying away a portion of the heat from the friction surface (a convective cooling-like effect). Upon examining the images of the wear surfaces of the specimens in Figure 13, wear lines forming in different directions are clearly visible. Furthermore, with the use of GGBS, it is evident that the contact area has increased compared to the other specimens due to the nearly seamless structure of the B25 series’ surface. These results are consistent with the surface roughness results. As a result, the GGBS replacement improves the abrasion performance of polymer concrete by 27%, while the increased surface contact area and material compactness increase the thermal load on the friction surface by approximately 10%.

### 4.6. Fracture Energy Results

**Figure 15:**
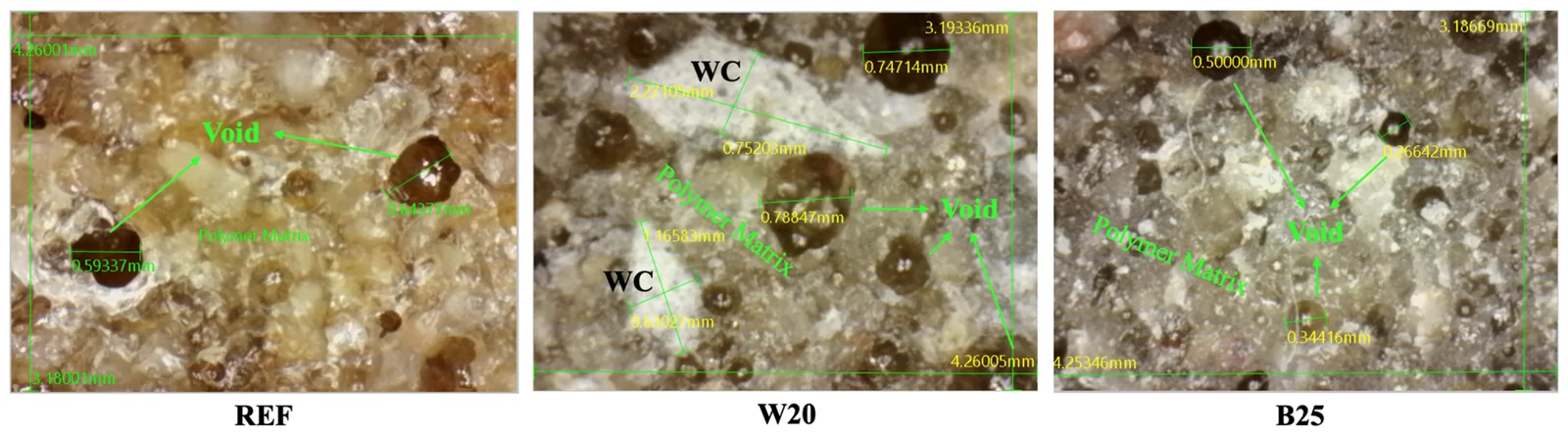
Digital microscope images of the specimens.

**Figure 16:**
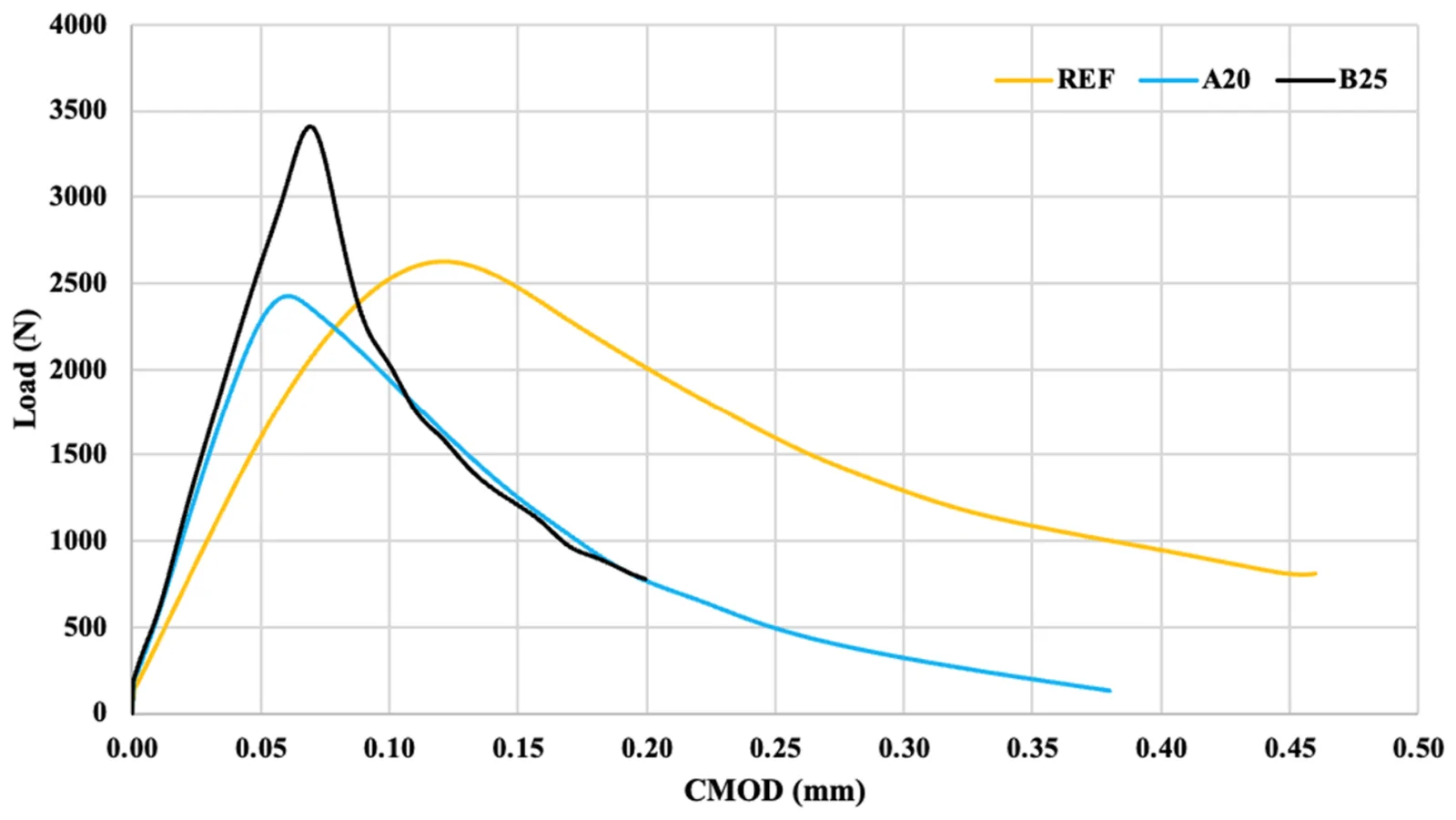
Load–CMOD responses of specimens.

**Figure 17:**
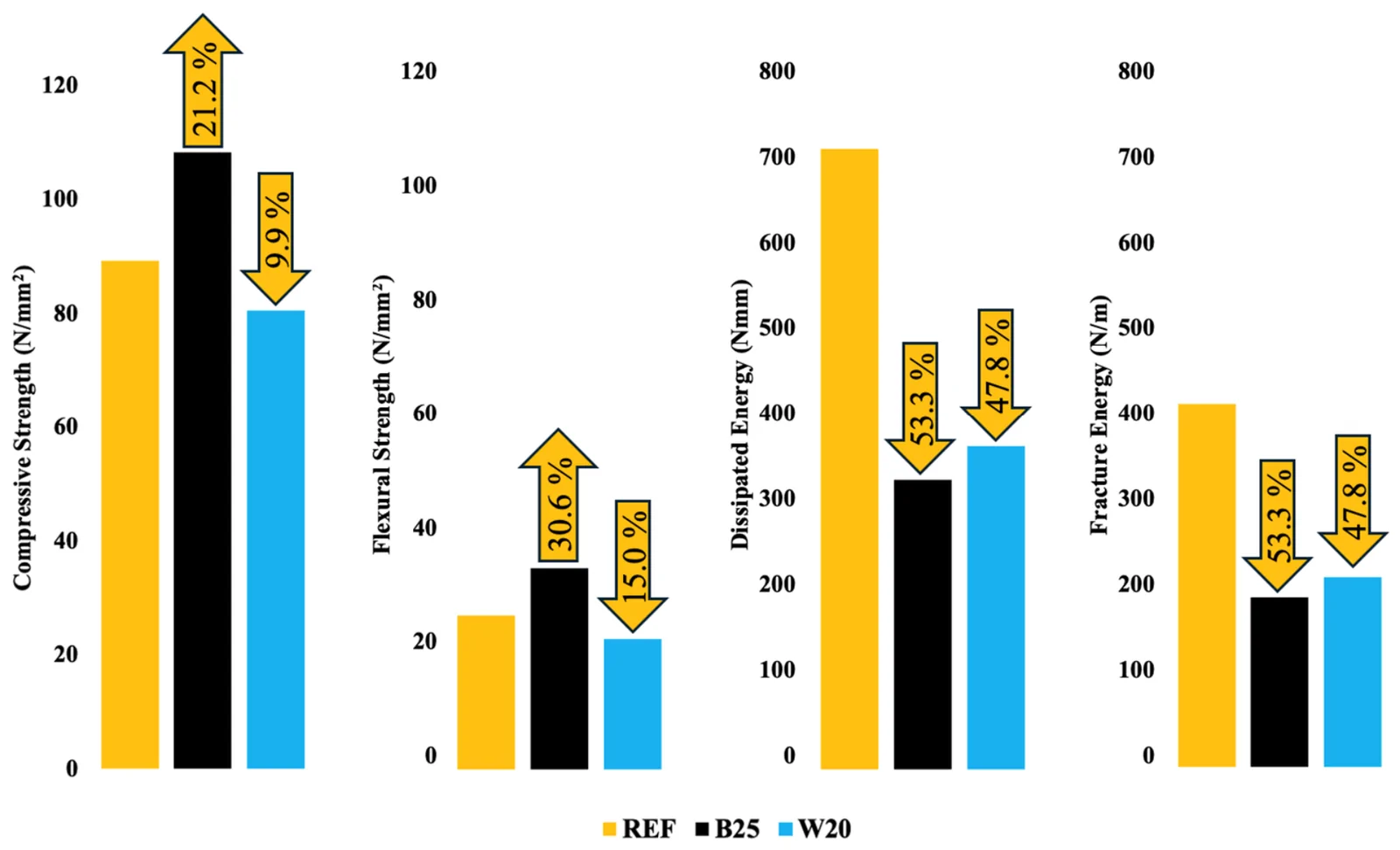
Fracture energy and dissipated energies of specimens.

Digital microscope images taken of the fracture surfaces of the specimens following the fracture energy test are shown in Figure 15. The load–CMOD curve obtained from the results of the fracture energy test carried out on notched specimens is shown in Figure 16, and the fracture energies (Gf) and total dissipated energies (Wf) are shown in Figure 17.

Upon examination of the fracture surface of the reference specimen (Figure 15), it is observed that the aggregates exhibit high adhesion to the matrix. The texture on the matrix surface is far from the mirror-like smoothness typically seen in brittle materials. At the micro level, “roughness” and deformation marks indicating polymer elongation are visible on the surface. This micro-roughness maximizes the material’s toughness by increasing the unit energy required for damage propagation at the moment of fracture. Additionally, air voids formed in the polymer matrix are observed in some areas. In the W20 series, more pronounced micro-voids and air voids are observed within the matrix structure compared to the reference and B25 series. These voids act as focal points for stress concentrations during the loading process. This is one of the reasons for the decrease in mechanical properties. The irregular shape, porous structure, and dimensions of the WC, which has replaced silica sand, are seen in detail in the figure. The overall topography of the fracture surface of sample B25 appears much flatter than the reference sample. This flatness suggests that the crack encountered little resistance as it progressed and that the material fractured rapidly by following the geometrically shortest path without being able to dissipate the energy. The crystalline structures and the fact that the matrix has penetrated every point of the aggregate indicate that the GGBS is more tightly and seamlessly encapsulated by the polyester resin. Upon close inspection, there are virtually no voids where the aggregates have detached. Instead, it is observed that the aggregates fracture in a single plane along with the matrix. This indicates that the aggregate and matrix work in harmony in terms of strength.

The B25 series is the polymer concrete mixture that performs the best by a wide margin in terms of both compressive (108.3 MPa) and flexural (35.29 MPa) strength. Compared to reference samples, there is an approximate 21% increase in compressive strength and a 30% increase in flexural strength. However, when considering the results for energy dissipation and fracture energy, this increase in strength has had a negative impact on the material’s ductility. The fact that its fracture energy is the lowest (190.60 N/m) indicates that this series is very rigid but has a weak energy dissipation capacity to stop a crack once it forms. In other words, although B25 has a high carrying capacity, it is prone to sudden fracture.

Although the reference specimen ranked in the middle in terms of strength properties, it performed approximately twice as well as the others in terms of dissipated energy (714.32 Nmm) and fracture energy (408.20 N/m). This situation proves that the bond (interface) between the reference matrix and the aggregates provides the best bridging effect during crack propagation. The material is capable of undergoing greater deformation than the other series before fracturing and can distribute the energy within the matrix.

The W20 series, on the other hand, exhibited a significant decrease compared to the reference material, both in terms of strength values (approximately a 10% loss in compressive strength and a 15% loss in flexural strength), fracture energy, and energy dissipation capacity. It can be concluded that the WC replacement leads to a weakening of the matrix structure or a reduction in the bond strength between the aggregate and the matrix. The fact that the dissipated energy decreased to 372.73 Nmm and the fracture energy to 213.0 N/m indicates that this series does not offer the same toughness as the reference. Similar studies have also shown that polymer concrete produced without fibers exhibits a brittle structure, and that the fracture energies are consistent with the results obtained [29].

In conclusion, when the three series were evaluated against one another, it was found that while the B25 series stands out in applications requiring high strength, the reference series demonstrates structural superiority over the other two series under loading conditions that demand energy dissipation capacity and post-crack ductility. To eliminate the brittle behavior in the B25 series and enhance toughness, reinforcing the matrix structure with fibers is proposed as an optimization strategy consistent with similar studies in the literature [30,31,32,33].

## 5. Conclusions

In this research, the physical, mechanical, and tribological properties of polyester-based polymer concrete (PC) were experimentally investigated, and the following key conclusions have been drawn:

- The obtained experimental results clearly demonstrated that the type of filler used in polymer concrete mixtures is a determining factor for both physical, mechanical, and tribological performance.
- At the end of the 1-, 7-, 14-, and 28-day water absorption tests, it was determined that none of the PC samples absorbed water.
- According to the unit weight results obtained from all samples, it was determined that the unit weights ranged from 1.83 g/cm^3^ to 2.06 g/cm^3^.
- As the GGBS replacement ratio increased, the compressive strength rose significantly from 82 MPa to 108 MPa, and the flexural strength increased from 28 MPa to 35 MPa.
- In the samples containing WC, the compressive strengths remained stable in the range of 77–80 MPa, while the flexural strength decreased as the additive content increased, dropping to as low as 22 MPa.
- When a 25% GGBS substitution (B25) was applied, it was found that the surface roughness (Ra) value decreased by approximately 72% compared to the reference value, thus maximizing surface quality.
- The Bohme abrasion test revealed that the W20 series had the highest volume loss at 4.661 cm^3^, while the B25 series exhibited the highest resistance with the lowest volume loss at 3.058 cm^3^.
- GGBS replacement improves the abrasion performance of polymer concrete by 27%, while the increased surface contact area and material compactness increase the thermal load on the friction surface by approximately 10%.
- Shore D measurements taken after the sulfuric acid test revealed a maximum decrease of approximately 10% in the B25 series and approximately 12% in the W25 series.
- The greatest decrease in compressive strength following the acid test occurred in the B15 series (approximately 23%).
- The W20 series exhibited a significant decrease compared to the reference material, both in terms of strength values, fracture energy, and energy dissipation capacity.

When all the results of the study are evaluated comprehensively, it can be concluded that the B25 series demonstrates the optimum performance for applications requiring high strength, despite exhibiting a more brittle structure The reference series exhibits average strength properties compared to the other two series, has a higher energy dissipation capacity, and may exhibit ductile behavior. On the other hand, although the substitution with WC causes a decrease in mechanical properties, it is considered suitable for applications with lower strength targets. In addition, the fact that the WC series is the most resistant to sulfuric acid testing may be a reason why it is preferred in industrial applications. From an environmental perspective, the results indicate that the newly designed mixtures will contribute to the utilization of waste and industrial by-products.

## Figures and Tables

**Table 1:** Properties of polyester resin.

Chemical Structure	*Orthophthalic*	Density	1.16 ± 2 g/cm^3^	Viscosity	450 ± 50 (cps)
Tensile Strength	65 ± 6 MPa	E Modulus (Tensile)	3 ± 0.3 GPa	Tmax	150 ± 10 °C
Flexural Strength	120 ± 10 MPa	E Modulus (Flexural)	2.7 ± 0.3 GPa		

**Table 2:** Test specimen dimensions.

Test	Specimen Dimensions (mm)
Unit Weight	40 × 40 × 160
Shore D	
Flexural Strength	
Surface Roughness	
Compressive Strength	40 × 40 × 40
Acid Resistance	
Water Absorption	
Bohme Abrasion Test	71 × 71 × 71 (±1.5)
Fracture Energy Test	50 × 50 × 240 (Notched Beam)

**Table 3:** Unit weight, compressive strength, and flexural strength test results.

Specimen Code	Unit Weight (g/cm^3^)	Compressive Strength (N/mm^2^)	Flexural Strength (N/mm^2^)
REF	1.91	89.32	27.02
B5	1.84	82.12	28.42
B10	1.87	88.18	30.27
B15	1.97	96.12	30.77
B20	2.04	105.82	33.71
B25	2.06	108.30	35.29
W5	1.83	77.00	24.22
W10	1.85	77.87	24.01
W15	1.88	79.69	23.06
W20	1.93	80.46	22.97
W25	1.93	80.17	22.08

**Table 4:** Bohme abrasion test result.

	REF	W20	B25
Volume Loss (cm^3^)	4.188 cm^3^	4.661 cm^3^	3.058 cm^3^

## Data Availability

The original contributions presented in this study are included in the article. Further inquiries can be directed to the corresponding author.
